# A lifestyle screening tool for young children in the community: needs and wishes of parents and youth healthcare professionals

**DOI:** 10.1186/s12913-024-10997-y

**Published:** 2024-05-03

**Authors:** Anne Krijger, Lieke Schiphof-Godart, Caren Lanting, Liset Elstgeest, Hein Raat, Koen Joosten

**Affiliations:** 1 Department of Neonatal and Paediatric Intensive Care, Division of Paediatric Intensive Care, Erasmus MC - Sophia Children’s Hospital, PO box 2060, Rotterdam, 3000 CB the Netherlands; 2 Department of Public Health, Erasmus University Medical Center Rotterdam, Rotterdam, the Netherlands; 3 Department of Medical Informatics, Erasmus University Medical Center Rotterdam, Rotterdam, the Netherlands; 4 Netherlands Organisation for Applied Scientific Research TNO, unit Healthy Living, Child Health expertise group, Leiden, the Netherlands; 5 Reinier Academy, Reinier de Graaf Hospital, Delft, the Netherlands

**Keywords:** Lifestyle, Toddlers, Youth healthcare, Prevention, Focus groups

## Abstract

**Background:**

Youth healthcare has an important role in promoting a healthy lifestyle in young children in order to prevent lifestyle-related health problems. To aid youth healthcare in this task, a new lifestyle screening tool will be developed. The aim of this study was to explore how youth healthcare professionals (YHCP) could best support parents in improving their children’s lifestyle using a new lifestyle screening tool for young children.

**Methods:**

We conducted four and seven focus groups among parents (*N* = 25) and YHCP (*N* = 25), respectively. Two main topics were addressed: the experiences with current practice of youth healthcare regarding lifestyle in young children, and the requirements for the lifestyle screening tool to be developed. The focus groups were recorded, transcribed verbatim and analysed using an inductive approach.

**Results:**

Both parents and YHCP indicated that young children’s lifestyles are often discussed during youth healthcare appointments. While parents felt that this discussion could be more in-depth, YHCP mainly needed clues to continue the discussion. According to parents and YHCP, a new lifestyle screening tool for young children should be easy to use, take little time and provide courses of action. Moreover, it should be attractive to complete and align with the family concerned.

**Conclusions:**

According to parents and YHCP, a new lifestyle screening tool for young children could be useful to discuss specific lifestyle topics in more detail and to provide targeted advice.

**Supplementary Information:**

The online version contains supplementary material available at 10.1186/s12913-024-10997-y.

## Background

In early childhood (0–4 years), unhealthy lifestyle behaviours, such as poor dietary intake, limited physical activity, excessive screen time and insufficient sleep, have been associated with adverse health outcomes [1–7]. Overweight and obesity are among the most prominent manifestations of these unhealthy behaviours [8]. According to the WHO, 5.1% of all children under five were overweight or obese in 2020 [9]. In the Netherlands, the prevalence of overweight (including obesity) in children aged 2–9 years was 15.5% in 2021; the prevalence of obesity was 4.8% [10]. These prevalence rates illustrate the large scale of current lifestyle behaviour problems. As lifestyle habits are formed early in life and may persist over time, lifestyle interventions in the early years hold the greatest potential for long-term health benefits [11, 12].

In the Netherlands, preventive youth healthcare is a free service that aims to promote, protect and secure the health, growth and development of children up to the age of 18 [13]. From birth onwards, all children and their parents are offered regular consultations, vaccinations and counselling at local child health clinics. Youth healthcare professionals (YHCP) work in multidisciplinary teams and can refer to specialized care when needed. Among the core activities of YHCP is screening, for example for unhealthy lifestyle behaviour. By identifying unhealthy lifestyle behaviour in children, YHCP can provide targeted advice to parents to help them improve their children’s lifestyles. As up to 95% of young children are reached by Dutch youth healthcare, this provides an excellent setting for lifestyle improvement and prevention of adverse lifestyle-related health consequences [14]. In practice, however, lifestyle screening in young children appears to be complex and time-consuming, and no unambiguous screening method or tool is available.

In 2018, the Dutch Ministry of Health, Welfare and Sport published the National Prevention Agreement, which describes policies to tackle overweight, smoking and problematic alcohol use [15]. This agreement called for the development of a screening tool that would provide insight into the lifestyle of children aged 0–4 years and give parents practical support in mitigating the long-term risks of an unhealthy lifestyle. In previous phases of the project, we studied existing lifestyle screening tools for children and examined bottlenecks and patterns lifestyle behaviours in Dutch toddlers [16–18].

Linking to current work practices in youth healthcare and alignment with the needs and wishes of parents and YHCP may be critical to the ultimate success of the new lifestyle screening tool. To this end, we carried out a target group analysis before actually developing the new tool [19]. The aim of this paper is to describe (1) current practice of youth healthcare regarding lifestyle in young children, and (2) the requirements for the lifestyle screening tool under development, according to parents and YHCP.

## Methods

### Study design

We conducted focus groups among parents of children aged 0–6 years and YHCP working in Dutch youth healthcare. The use of focus groups allows for interaction between participants, which may lead to additional insight into the topics discussed [20]. Prior to the focus groups, participants completed a questionnaire assessing general characteristics. For the parents, this concerned their age, sex, education level, country of birth, number of children and age of their children. From YHCP, their profession (youth physician or youth nurse) and the healthcare centre they were appointed at were obtained. This study is reported as indicated by the COREQ (COnsolidated criteria for REporting Qualitative Research) Checklist (Additional File 1) [21].

### Participants

Participants were recruited using convenience and purposive sampling between April and October 2021. Parents were able to sign up via a previously conducted survey that served as a first exploration of the topic of lifestyle among parents of young children. In addition, parent recruitment leaflets were posted at youth healthcare centres, nurseries and the Erasmus University Medical Centre. Personal networks of members of the research team were also contacted and snowball recruitment occurred through parents who had signed up. The inclusion criteria for parents were: (1) having at least one child between the ages of 6 months and 6 years, and (2) being able to provide informed consent. There were no exclusion criteria. As data collection took place, we noticed that parents with lower educational attainment and parents with a migrant background were under-represented. A final recruitment attempt was therefore made through ‘parent contact persons’ at schools in Rotterdam, the Netherlands, with a relatively large population of low-educated families and families with a migration background.

YHCP (both youth physicians and youth nurses) working with children between the ages of 6 months and 6 years were eligible for inclusion. They were recruited through the research team’s professional network and through *JGZ Life!*, an online current affairs program for professionals within youth healthcare.

### Data collection

Data collection was performed in Dutch between June and November 2021 and took place for parents and YHCP separately. Participants could indicate their availability on predefined time slots. We tried to have between four and eight participants per focus group, but twice we accepted that there would be two participants in a focus group and once three. Due to COVID-19 measures, the first focus groups were held online via MS Teams. However, at the end of 2021, COVID-19 measures were loosened and we were able to conduct the focus groups with lower-educated parents and parents with a migration background in dedicated parent rooms at their children’s schools. In addition to the information letter and written informed consent, the focus group moderator briefly explained the study aims and participants verbally reconfirmed their consent to audio recording, at the beginning of each focus group. During the focus groups, at least two members of the research team (CL (MD, PhD, extensive focus group experience), MdW (PhD, extensive focus group experience), LSG (PhD, extensive focus group experience), and AK (MD), all female researchers) were present and field notes were taken. The ‘parent contact persons’ were also present at the focus groups in the schools. We developed separate topic guides for parents and YHCP. The key questions for both parents and YHCP in this topic guide concerned: 1) the current practice of youth healthcare regarding young children’s lifestyle, and 2) the requirements for the lifestyle screening tool under development. Prior to topic two, the moderator summarized the main idea of the new lifestyle screening tool (i.e. asking parents questions regarding their children’s lifestyle preceding a youth healthcare visit, leading to tailored advice). The lifestyle screening tool had not been mentioned to the participants before, in order to avoid narrowed data collection for the first topic. All participating parents received a gift card as a token of appreciation; YHCP received an attendance fee.

### Data analysis

All audio recordings were transcribed verbatim. The transcripts were coded using NVivo software (QSR International Pty Ltd. (2022) Nvivo (Release 1.7)) and analysed using an inductive thematic approach [22]. First, two researchers (AK and KK) openly coded two transcripts independently, one from parents and one from YHCP. These preliminary coding schemes were compared, refined and discussed with LSG until consensus about the axial coding framework was reached. Next, AK and KK coded the remaining transcripts. In consultation, new codes were added to the coding scheme and AK and KK checked the consistency of all coded transcripts. AK and LSG agreed that data saturation was achieved. Through a process of discussion, agreement was reached on overarching themes and key findings of the data. Descriptive characteristics of the study samples were summarized using Microsoft Excel 2016.

## Results

### Participant characteristics

Seven focus groups were held among parents and four among YHCP. The average durations were 58 and 56 min for parents and YHCP, respectively. The characteristics of participating parents and YHCP are given in Table 1. The average age of parents was 38.0 years (SD 4.4, range 31–46) and the majority were female (96%). The mean number of children per parent was 2.6 (SD 1.7, range 1–7). Most of the parents had been born in the Netherlands (64%) and had received a high level of education (44%). Five YHCP dropped out because the scheduled time did not suit them. Participating YHCP were predominantly female (96%). Most of them were working as a youth physician (68%) and the largest portion in the Western part of the Netherlands (48%).

**Table 1:** Characteristics of participating parents and YHCP

	**Parents (** ***N*** ** = 25)**
**Age (years)** ^a^	38.0 (31–46)
**Gender (%)**	
Female	96
Male	4
**Number of children**	2.6 (1–7)
**Country of birth (%)**	
The Netherlands	64
Morocco	32
Tunisia	4
**Educational level (%)** ^**b**^	
Low	20
Middle	36
High	44
	**Youth healthcare professionals (** ***N*** ** = 25)**
**Gender (%)**	
Female	96
Male	4
**Profession (%)**	
Youth physician	68
Youth nurse	32
**Region in the Netherlands (%)**	
North	4
East	28
South	20
West	48

Values are mean and range or percentages. ^a^One missing on age. ^b^Low, primary education and pre-vocational secondary education; middle, higher secondary vocational education; high, higher professional education and university education

### Current practice of youth healthcare regarding young children’s lifestyle

#### Parents

Regarding the current practice of youth healthcare regarding young children’s lifestyles, the themes that arose were: (1) screening and discussing lifestyle, and (2) advising and informing. Parents stated that their child’s lifestyle is often discussed during youth healthcare appointments and that they appreciate this. The emphasis of the conversation is usually on nutrition, but the topics of physical activity and sleep are also commonly addressed. Parents value the open-ended, non-judgmental questions asked by YHCP to start the conversation. However, when asked to clarify their preferences, parents expressed that YHCP could ask more in-depth questions, such as how much the child is eating exactly or what the vegetable intake is like. According to the parents, this may provide YHCP with a better understanding of the situation from which to offer specific advice. It may also help to break down barriers that might prevent parents from sharing their concerns if only open-ended questions are asked.

Regarding parents’ preferences for screening and discussing lifestyle:

*“If a child is growing well and following the curve, then it’s basically done. But you [the YHCP] could also zoom in on what they [the children] actually eat and what the fruit and vegetable intake is like.”* Parent #5.

Moreover, parents indicated that the lifestyle conversation could be more in line with their needs and family situation. Barriers to discussing lifestyle, according to parents, are the relatively few appointments offered by youth healthcare services and time constraints. Some parents put forward that not all YHCP were equally open to alternative ways of eating or upbringing.

Parents reported receiving advice and information about their child’s diet, physical activity and screen use. In general, parents were satisfied with the advice they received. However, the advice and information was also repeatedly perceived as not being very comprehensive and not giving enough guidance on what is healthy. As facilitators in informing about lifestyle by YHCP, parents reported explaining guidelines and advice and providing information material to take home.

With regard to the way information is given, parents prefer a coaching, non-strict conversation with a holistic perspective. The presence of older children in the family is a major obstacle in compliance with lifestyle advice for their young children. In the focus groups with parents with lower levels of education and migrant backgrounds, the grandparents’ views on a healthy lifestyle and a healthy weight were also noted as disturbing factor.

Regarding the perception of older family members as a barrier to complying with lifestyle advice:

*“When I go on holiday to my family, they say: ‘Oh he is cute, but skinny, so sad’.” [when in fact he has a normal weight]* Parent #13.

#### YHCP

For YHCP, the themes on the current practice of youth healthcare regarding young children’s lifestyles that arose: (1) screening and discussing lifestyle, and (2) advising and informing. YHCP indicated that the subject of lifestyle is discussed in the majority of appointments. Exceptions include appointments on indication, for example when vision or motor skills are examined only. When children are younger than one year old, lifestyle, particularly nutrition, is often addressed at the parent’s initiative. Parents may have questions themselves, and also expect talking about their child’s nutrition. After the first year of life, parents typically bring up the topic of nutrition only when they experience problems, such as the child not eating well or being a picky eater. YHCP stated that if parents do no mention lifestyle themselves, they will inquire about it as openly as possible.

Regarding the current method of screening and discussing lifestyle during a youth healthcare visit:

*“Well, I basically just ask at every consultation: ‘How is the diet?’. And then we talk about that.”* YHCP #5.

In addition to nutrition, YHCP may discuss with parents their children’s physical activity, screen time, sleep, as well as family stressors, parenting and parental lifestyle. Sometimes this conversation is initiated on the basis of a child’s growth curve or specific items in the electronic health record, such as supplemental vitamin D intake. Several YHCP also mentioned that tools, such as a waiting room poster that displays the number of sugar cubes in various sugar-sweetened beverages, frequently spark discussion. However, the demand-driven way of working within Dutch youth healthcare and time constraints make it sometimes challenging to discuss lifestyle with parents, especially when YHCP feel there are no “starting points”, such as unhealthy weight, for the conversation.

Regarding the absence of “starting points” to proceed with the conversation:

*“So, when I ask ‘How is the diet?’, and the answer is ‘Good’, yes, then it gets difficult. Because indeed, how much further should you ask? If I see a child having overweight or obesity, then I really have a starting point for a conversation, but when I see a child with a healthy weight who is developing well, yes… Then I’ll let it go, then I won’t ask any further questions. So, I’m probably missing a lot of things.”* YHCP #7.

Regarding time constraints during consultations:

*“You have several things to do and this [discussing lifestyle] is just a small part of it. In that respect, I believe I absolutely miss children who may have an unhealthy diet but are otherwise healthy-weighted. But because you just have 20 minutes and there are so many things you need to give attention to, that goes wrong sometimes.”* YHCP #14.

Nevertheless, when YHCP notice “red flags”, such as abnormal growth or overweight, they probe further. While it is easier to start the conversation about lifestyle in this case, YHCP find it more difficult to continue this conversation. Reasons for this are mainly parent-related: some parents may find the topic of lifestyle too sensitive, they may not be open to a conversation about it, or are unaware of the lifestyle recommendations for a specific age.

With regard to advising and informing parents about lifestyle of their children, YHCP indicated a list of facilitators and barriers. Above all, it was stated that advice or information given should be tailored to the family concerned. To facilitate this, YHCP reported that provided advice and information should be in line with the parents’ knowledge, skills, financial resources, environment, and culture. Additionally, using existing tools and information sources, such as flyers from the Dutch Nutrition Centre, and offering feasible advice was considered helpful. Most barriers were related to these facilitators. In addition, the resistance of parents to advice was also raised as a major concern.

Regarding parental resistance to lifestyle advice:

*“But here again, if parents notice that their child is overweight but refuse to do anything about it, it is better to ask parents again when they begin to worry about it. (.) However, it gives me mixed feelings, because the child has no choice. (.) So, I still find that very difficult.”* YHCP #16.

### Requirements for a new lifestyle screening tool

#### Parents

**Fig. 1:**
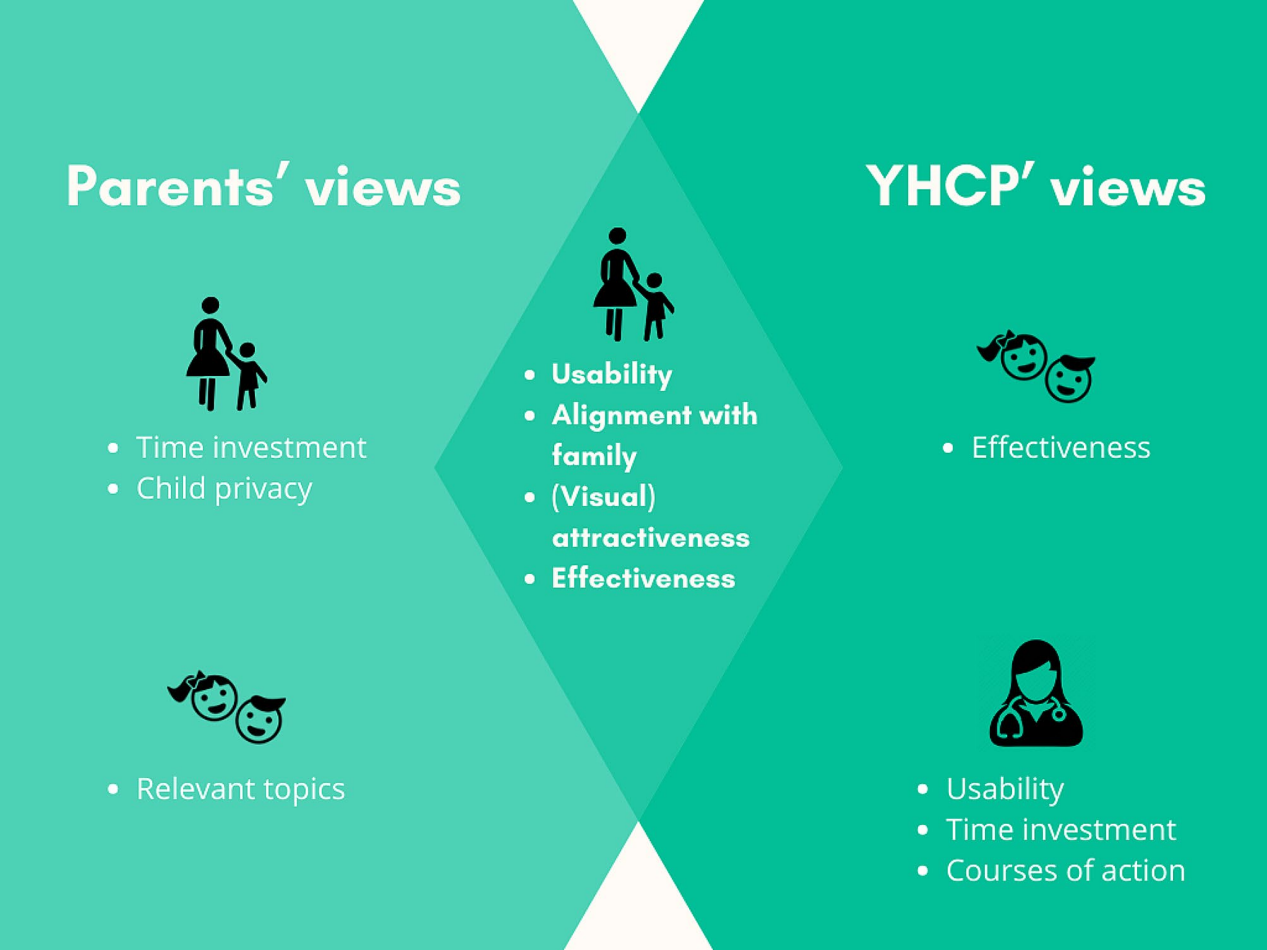
Overlapping and individual themes that emerged for parents, children and YHCP according to parents and YHCP

The requirements that emerged from the parents were divided into requirements for themselves and for their children (Table 2; Fig. 1). Six themes were identified in terms of requirements for the parents themselves: (1) usability, (2) time investment, (3) alignment with family, (4) visual attractiveness, (5) effectiveness, and (6) child privacy. Usability mainly concerned completing the tool at a suitable place (e.g. at home or waiting room) and in a practical way (digitally or on paper). Although opinions varied on the best place and method, parents agreed that the time investment should be minimal and certainly no longer than ten minutes. To align the lifestyle screening tool with the family, parents requested that the tool be tailored to the family’s needs and values in terms of socio-economic status, skills and family culture. Parents preferred a visually appealing tool that provides an overview of a child’s lifestyle.

Regarding effectiveness and visual attractiveness:

*“And that’s why I thought of a spider web, because then you can show the relationship between the different elements, and as professional you can also say: ‘Hey, I’m noticing something here’.”* Parent #2.

As for effectiveness, major concerns for parents were that the purpose of the tool should be clear to them and that YHCP act upon the answers parents provide. Moreover, the tool should mainly facilitate and support the conversation with the YHCP and not be strict and patronizing. While the higher-educated parents emphasized the importance of using the tool holistically and without judgment, the parents with a lower education and/or migration background indicated that they would prefer outcomes with more direction. The use of a traffic light system, for example, in which healthy behavior is marked green and less healthy behavior orange or red, would give them guidance and motivation to improve.

Regarding outcomes with a clear direction as part of the theme ‘effectiveness’:

*“Of course! When I get a warning like ‘your child can do much better’ (…), you just do your best!”* Parent #14.

Some parents mentioned that a tool would have been helpful before the age of one, whereas others stated that they had more questions during toddlerhood and such a tool would therefore be more effective from the age of 12 months and older. Finally, parents considered it critical to ensure the safety of the data they would provide with the tool.

The requirement for the child comprised including relevant topics in the tool. The parents suggested nutrition, physical activity and sleep as the most relevant topics. Screen time was not mentioned.

#### YHCP

YHCP devised requirements for the new lifestyle screening tool for themselves, for the parents, and for the children (Table 2; Fig. 1). As for requirements for YHCP themselves, three themes were identified: (1) usability, (2) time investment, and (3) courses of action. Usability referred to several factors, including using the tool as a conversation aid, embedding it into the current working method and electronic health record, and utilizing existing tools and resources for providing advice and information. Regarding time investment, the most frequently mentioned concern for YHCP themselves was that the instrument should not lead to time loss during the appointment. Lastly, the YHCP mentioned that the tool should offer them courses for action, for example by providing a score, offering cues for the conversation or contributing to counselling.

Regarding using the tool as a conversation aid:

*“Could it be a starting point for the conversation you are already having anyway, but in a certain way, from that starting point?”* YHCP #2.

According to the YHCP, the requirements for the parents were subdivided into: (1) usability, (2) alignment with family, (3) attractiveness, and (4) effectiveness. YHCP expressed that the tool should have high usability for parents too, for example by enabling quick and digital completion. In addition, the YHCP above all felt that a new lifestyle screening tool should align with the family, particularly in terms of the parents’ needs, socio-economic status, skills, and culture. Other requirements for parents for the tool included it being attractive, i.e. visually appealing and not too strict or patronizing, as well as being effective, for example by increasing parents’ knowledge and awareness of their child’s lifestyle.

The overarching theme of the requirements for the children according to YHCP was effectiveness. YHCP mentioned that a new lifestyle screening tool would be effective for children if it covers relevant topics and is used at appropriate ages. Healthy and unhealthy dietary intake and physical activity were most frequently mentioned as relevant topics, but screen time, sleep and smoking also emerged. YHCP agreed that a lifestyle screening tool should be applied before lifestyle patterns become ingrained, so for example at the age of one year, or even earlier.

**Table 2:** Summarized requirements for a new lifestyle screening tool according to parents and YHCP

	Parents’ views		YHCP’ views	
Target group	Themes	Requirements	Themes	Requirements
**Parents**	• Usability	Tool completion at home or in waiting room; digitally or on paper	• Usability	Quick and digital tool completion
	• Time investment	Completion takes no longer than ten minutes		
	• Alignment with family	Tool takes into account parental needs, socio-economic status, skills and culture	• Alignment with family	Tool takes into account parental needs, socio-economic status, skills and culture
	• Visual attractiveness	Visually appealing tool that provides an overview	• Attractiveness	Visually appealing tool that is not strict or pedantic
	• Effectiveness	Tool purpose should be clear; YHCP should act upon answers given; tool should support an open conversation; tool application from age of one year	• Effectiveness	Tool should increase parents’ knowledge and awareness
	• Child privacy	Data security should be ensured		
**Children**	• Relevant topics	Nutrition, physical activity, sleep	• Effectiveness	Topics: healthy and unhealthy dietary intake, physical activity, screen time, sleep, smoking; tool application from age of one year or earlier
**YHCP**			• Usability	Tool should support conversation, be embedded in current working method and electronic health record and use existing resources for providing advice and information
			• Time investment	Tool usage should not lead to time loss during appointment
			• Courses of action	Tool should offer a score or cues for the conversation or counselling

## Discussion

This study describes the experiences of current practice in Dutch youth healthcare regarding lifestyle in young children and the requirements for a new lifestyle screening tool according to parents and YHCP. A new lifestyle screening tool was considered desirable by both groups.

Parents reported that they were generally satisfied with the current practice in youth healthcare regarding the lifestyle of young children. They appreciated the open start to the lifestyle conversation, but required a more in-depth approach from the YHCP, both in continuing the conversation and in providing advice and information. This finding is in line with Swedish research in which parents indicated the desire to receive more information and advice regardless of their identified needs [23]. Parents in our study felt that this could be overcome by further questioning on specific lifestyle topics, and also by providing more explanation and background to the guidelines and advice, or by offering information materials to take home. Asking specifically about the habitual quantity of fruit consumption or hours of screen time, for example, may not be in line with the demand-driven approach used in Dutch youth healthcare. A common conversation technique within this demand-driven methodology starts from the parents’ concerns in order to actively engage them in the conversation [24]. This technique is based on the idea that care can then be tailored to parents’ needs and that parents will be more motivated to make changes if they themselves perceive certain issues as problems. YHCP also experienced that sometimes they want to continue a conversation about lifestyle with the parent, but they lack “starting points” or tools to do so. In their view, the demand-driven approach then conflicts with the need to work preventively. A lifestyle screening tool could address this concern by first asking an open-ended question about the parent’s perspective and then eliciting more specific information about certain lifestyle topics. In this way, both the parent and the YHCP are given a helping hand to guide and deepen the conversation, discuss topics that might not otherwise be covered, and allow the parent to get specific advice.

Although the nuance of the themes was slightly different for parents and YHCP, we found considerable overlap between the requirements of both groups. Above all, for parents as well as YHCP, a new lifestyle screening tool for young children should be easy to use, take little time, and provide concrete courses of action. Furthermore, for parents, tool usage should align to the family in question and be (visually) attractive to use. In our view, these requirements may also be relevant to other innovations within youth healthcare. Support that matches personal experiences, preferences and practices that is culturally sensitive was also expressed as a need in a Dutch study that examined parents’ perspectives regarding youth health care in the first two years [25]. For the lifestyle screening tool, for example, this could mean that the advice given by the YHCP takes into account the family’s food culture and financial resources. In another Dutch study on psychosocial and lifestyle assessment of childhood obesity, care professionals also stated that visual materials are helpful in conversations with parents [26].

Higher-educated parents and YHCP felt that screening tool outcomes should not be too judgmental, whereas parents from less educated or migrant backgrounds needed more clarity in the answers given and were open to a more directive approach. As children of parents with lower education levels or migrant backgrounds are more likely to have unhealthy lifestyles, there is more to be gained especially there [27, 28]. A ‘traffic light’ system indicating healthy and unhealthier behavior, as suggested by these parents, may therefore be a useful, clarifying and effective feature of the new lifestyle screening tool.

While focus groups are used to gain in-depth knowledge of people’s perceptions, beliefs and opinions about a particular (health) issue, they are also useful in identifying the target population’s needs and wishes when developing innovations [29–31]. Although most of the findings of other focus group studies are specific to the innovations, they all concluded that the use of focus groups supports the development itself and future acceptability [32–35].

### Strengths and limitations

Strengths of this study include conducting focus groups with both stakeholder groups, namely parents and YHCP. This qualitative approach allowed for the collection of enhanced opinions and data from person-to-person interactions, as well as comparison of the two groups [20]. Our efforts to reach more parents with a lower education level and non-Dutch background increased the transferability of our findings. Credibility was raised by the data being coded independently by two researchers. The use of convenience sampling in parent recruitment is a study limitation, which was partly due to COVID-19 regulations in place at the time. COVID-19 regulations also required most of the focus groups to take place online. While this may not have affected the data quality, face-to-face interviews may be preferable when discussing socially sensitive topics, such as lifestyle [36]. As most of the parents in our study had several children, we should be aware that the results may be different for first-time parents. Lastly, the limited involvement of fathers, the lack of diversity of cultural backgrounds and the limitation to Dutch-speaking parents should be taken into account when interpreting the results in the context of the Netherlands as a whole.

## Conclusions

Young children’s lifestyles are often discussed during youth healthcare appointments. While parents felt that these conversations could be more in-depth, YHCP needed resources to carry on the conversation. A lifestyle screening tool may be able to respond to these desires. According to parents and YHCP, this screening tool should be easy to use, take little time and offer courses of action. For parents in particular, the tool should be attractive to complete and align with the family in terms of parental needs, socio-economic status, skills and culture. These preferences need to be considered throughout the development of the new lifestyle screening tool to increase its effectiveness, acceptability and value in improving the lifestyle of young children. To reach the group that would benefit most from lifestyle improvements, i.e. families from lower socio-economic backgrounds, it is crucial to specifically address their needs.

### Electronic supplementary material

Below is the link to the electronic supplementary material.

Supplementary Material 1

## Data Availability

The datasets used and/or analysed during the current study are available from the corresponding author on reasonable request.

## Abbreviations

YHCP: Youth healthcare professionals
